# Prevalence and correlation of abnormal flow reserve by stress CMR and coronary artery plaque by cardiac CTA in symptomatic diabetics

**DOI:** 10.1186/1532-429X-16-S1-O57

**Published:** 2014-01-16

**Authors:** Yasmin S Hamirani, Michael Salerno, Yang Yang, Christopher M Kramer, Jamieson Bourque

**Affiliations:** 1 Cardiology, University of Virginia, Charlottesville, Virginia, USA; 2 Department of Biomedical Engineering, University of Virginia, Charlottesville, Virginia, USA; 3 Department of Radiology, University of Virginia, Charlottesville, Virginia, USA

## Background

Coronary artery disease (CAD) is a source of significant mortality and morbidity in diabetes mellitus (DM). Abnormal myocardial perfusion reserve (MPR) has been shown to predict worse outcome in patients with diabetes, but limited data is available using CMR. The correlation of MPR derived from Cardiac Magnetic resonance imaging (CMR) with plaque burden on cardiac computed tomography angiography (CTA) has not been previously evaluated. We aim to look at this correlation in patients undergoing vasodilator stress CMR.

## Methods

Same day Adenosine- stress CMR (Siemens Avanto 1.5 T) and cardiac CTA (Dual source 128 detector scanner) were performed on symptomatic diabetics without known coronary artery or valvular heart disease, referred for stress testing. Both qualitative and quantitative perfusion analysis (using Fermi-deconvolution in Matlab and 16 segment AHA model) were performed. Rest and stress myocardial blood flow and MPR were calculated. Patients were divided into three groups based on an MPR of < 2.0, 2 -2.4 and > 2.4. Semi quantitative assessment of plaque using CTA (plaque and segment scores) were calculated as published in the literature and compared using ANOVA and Tukey's analysis.

## Results

Amongst the 25 patients enrolled, the mean age was 60 +/- 9 yrs., 60% were females, and the mean BMI (kg/m2) was 31.7 +/- 6.56. No coronary plaque was visualized in 9 (36%) of the patients while two patients (4%) had a > 50% plaque in atleast once coronary artery. A qualitative perfusion abnormality was detected in 2 subjects (4%), one of whom had a > 50% plaque on CTA. Reduced flow reserves of < 2.0 and 2-2.4 were present in 5 (20%) and 9 (36%) of patients respectively, leaving 11 (44%) with normal MPR > 2.4. CTA findings as well as stress CMR- MPR analysis are presented in table 1. There was a stepwise increase in CTA plaque burden as measured by segment score with a substantial increase for MPR < 2.0. The plaque score increased significantly only for MPR < 2.0. No association of type of coronary plaque with MPR was detected.

**Table 1:** 

Characteristics	Total population (No. 25)	MPR < 2.0(No.5)	MPR:2-2.4 (No. 9)	MPR > 2.4 (No.11)	P value
CTA plaque score (max: 64)	5.48 ± 7.33	12.4 ± 11.76	3.44 ± 4.06	4 ± 5.51	0.05
CTA segment score (max: 16)	3.36 ± 4.43	8.2 ± 6.09	2.44 ± 3.81	1.9 ± 2.42	0.016
Presence of non-calcified plaque in any segment	2 (8%)	0	1 (11.11%)	1 (9.09%)	1
Presence of calcified plaque in any segment	15 (60%)	4 (80%)	5 (55.56%)	6 (54.55%)	0.75
Presence of mixed plaque in any segment	3 (12%)	1 (20%)	0	2 (18.18%)	0.5
Global MPR	2.55 ± 0.94	1.42 ± 0.347	2.10 ± 0.89	3.44 ± 0.63	0.00
Average global rest flow (ml/g/min)	0.687 ± 0.20	0.776 ± 0.310	0.654 ± 0.13	0.67 ± 0.19	0.54
Average global stress flow (ml/g/min)	1.698 ± 0.624	1.016 ± .53	1.37 ± 0.26	2.2 ± 0.43	0.00

MPR: Myocardial perfusion reserve; CTA: Cardiac Computed tomography angiography

## Conclusions

A reduced MPR < 2.4 was found in the majority of our cohort of symptomatic diabetic patients referred for stress CMR (56%), and 36% had a substantially-decreased MPR of < 2.0. A significant correlation between extent of plaque and MPR was identified in this population with a substantial increase with MPR < 2.0. Long-term follow-up will determine the prognostic utility of these findings.

## Funding

George Beller Research Award, an internal funding award from the Heart Center Advisory Board.

**Figure 1:**
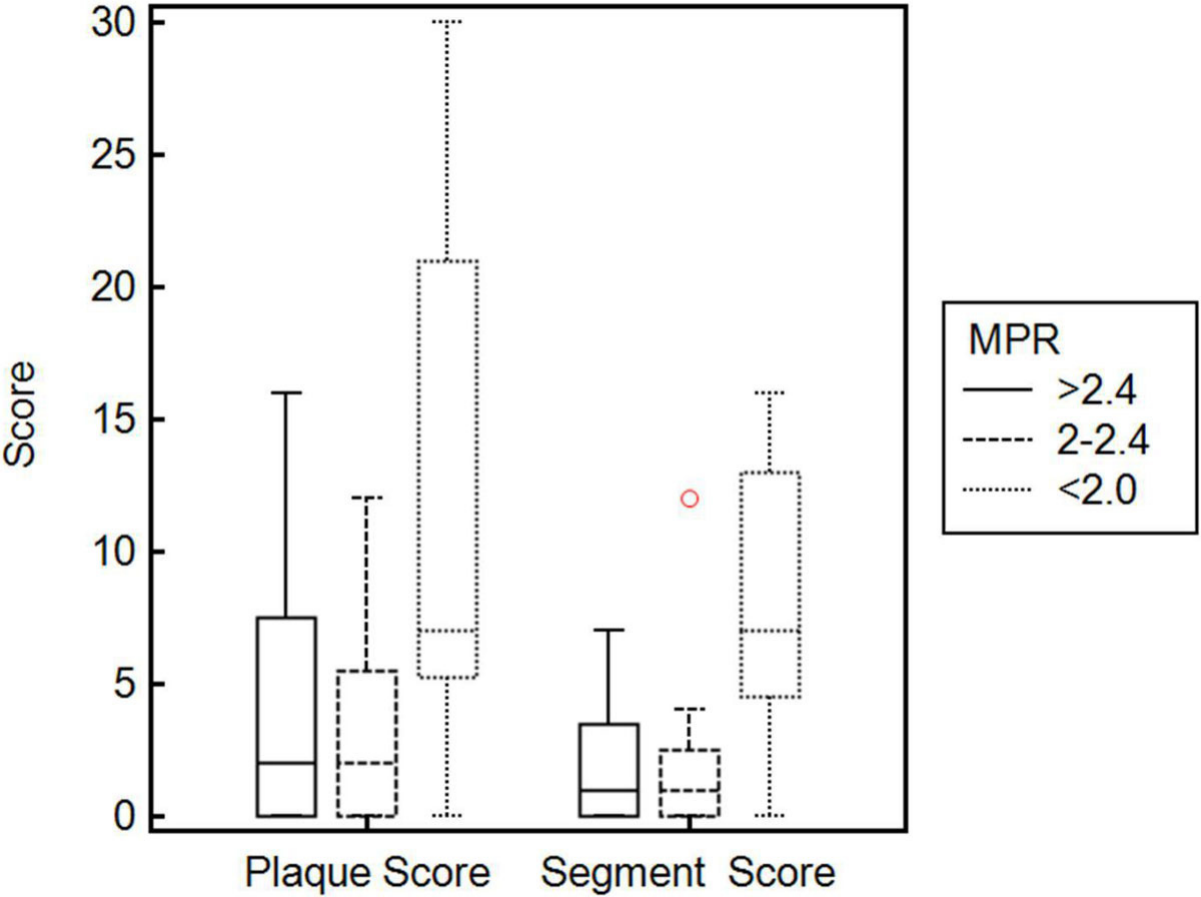
**CTA plaque score and segment score on patients with CMR-MPR < 2.0, 2-2.4 and > 2.4**. CTA: Cardiac computed tomography angiography; CMR: Cardiac Magnetic Resonance Angiography; MPR: Myocardial perfusion reserve.

